# Celastrol-conjugated chitosan oligosaccharide for the treatment of pancreatic cancer

**DOI:** 10.1080/10717544.2021.2018521

**Published:** 2021-12-29

**Authors:** Xiaohu Zeng, Xin Zhu, Qikang Tian, Xiaoke Tan, Ning Sun, Min Yan, Junwei Zhao, Xiangxiang Wu, Ruiqin Li, Zhenqiang Zhang, Huahui Zeng

**Affiliations:** a Academy of Chinese Medicine Sciences, Henan University of Chinese Medicine, Zhengzhou, China; b School of Basic Medicine, Henan University of Chinese Medicine, Zhengzhou, China; c Pharmacy College, Henan University of Chinese Medicine, Zhengzhou, China; d Henan Traditional Chinese Medicine Innovation Engineering Technology Research Center, Zhengzhou, China; e Department of Clinical Laboratory, The First Affiliated Hospital of Zhengzhou University, China Zhengzhou Henan

**Keywords:** Celastrol, Chitosan oligosaccharide, pancreatic cancer, toxicity, drug delivery system

## Abstract

Celastrol is a promising antitumor drug candidate, but the poor water solubility and cytotoxicity limit its clinical application. Herein, we synthesized a Celastrol (Cel)-chitosan oligosaccharide (CSO) conjugate (Cel-CSO) for drug delivery. Celastrol was conjugated to a CSO backbone via amide bond formation, which was verified by infrared spectrum (IR) analyses. The Cel-CSO contained ∼10 wt% of Celastrol showed excellent aqueous solubility (18.6 mg/mL) in comparation with the parent Celastrol. Cel-CSO significantly inhibited tumor growth, induced apoptosis, and effectively suppressed tumor metastasis in human pancreatic cancer cells (BxPC-3). While the cytotoxicity of Cel-CSO in hepatic cells (HL7702) was lower than that of the free Celastrol. Cel-CSO enhanced the anticancer efficacy, promoted the circulation time of Celastrol, and reduced the subacute toxicity, which indicated that CSO can be a promising Celastrol delivery system for pancreatic cancer therapy.

## 1. Introduction

Pancreatic cancer is one of the highest aggressive and lethal human malignancy with a 5-year survival rate of approximately 5%, which has a low percentage of 2% among all cancers but 5% of cancer deaths worldwide (McGuigan, 2018). Currently, gemcitabine still is the first-line chemotherapeutic drug for treatment of pancreatic cancer. Gemcitabine demonstrates a median survival advantage of 6 months versus 5 months for its predecessor 5-fluorouracil (5-FU) (Chugh et al. 2012; Giri et al. 2017). However, its clinical efficacy is not satisfactory, because the potential drug resistance allows pancreatic cancer to elude cell death under various survival mechanisms (Wang et al. 2016). To date, no other chemotherapeutic drug for pancreatic cancer is potential for promoting a clinically meaningful prolongation of overall survival. Hence, new therapy strategies are still urgent needs for the development of novel drugs to improve the therapeutic effect of pancreatic cancer.

Celastrol (Cel) is one of the principal bioactive ingredients derived from the roots of the Chinese medical herb *Tripterygium wilfordii*. Celastrol has a broad range of bioactivities against multiple complex diseases, such as cancer (Chen, 2020; Niu, 2020), rheumatoid arthritis (An et al. 2020), lupus erythematosus (Xinqiang, 2020), and Alzheimer’s disease (Bai et al. 2021). Recently, celastrol and its derivatives have attracted immense attention on their anticancer activities (Shan, 2019; Hou, 2020). Although celastrol has various pharmacological mechanisms in treating pancreatic cancer, the potentiality in clinical application is significantly restricted by its poor water-solubility and severe toxicity. For that, many strategies were used to solve the physicochemical limitation and dose-limiting toxicity of celastrol, such as polymeric prodrugs (Davenport et al. 2010), nanospheres (Zhang et al. 2014), and polymeric micelles (Peng et al. 2012; Shi et al. 2020). It has been reported that many celastrol-loaded carriers significantly ameliorate its bioavailability and therapeutic efficacy, but reduce theirs side effects (Huang et al. 2012; Qi et al. 2014; Zhang et al. 2014). However, those celastrol drugs have not yet entered the clinical application stage. Hence, there are still urgent needs to develop novel celastrol formulation for clinical use.

In recent years, the polymer-drug conjugates have been developed adequately for clinical application of the hydrophobic drug. First of all, the polymer-carrier materials must be nontoxic, low immunogenic and highly soluble in water, such as PHPMA (poly[N-(2-hydroxypropyl) methacrylamide]) (Zhang et al. 2018), polyethylene glycol (D’souza and Shegokar, 2016), and polysaccharide polymers (e.g. chitosan, dextran, etc.). PK1 (PHPMA copolymer doxorubicin) is the first member of drug-polymer conjugates in phase I clinical trial (Vasey et al. 1999). Afterwards, AD-70 (Dextran copolymer doxorubicin) was developed as the first dextran-drug conjugate to enter clinical trials. However, AD-70 shows some degree of hepatotoxicity, thrombocytopenia and other adverse symptoms, because dextran is easily ingested by the endothelial reticular system (Danhauser-Riedl et al. 1993). Xyotax^®^ (Paclitaxel-conjugated polyglutamate) is a clinical antitumor prodrug with a good pharmacokinetic profile (Zhao, 2018). Polyglutamate as a polymer carrier showed no toxicity and low immuno-genicity *in vivo*. However, the polyglutamate backbone from artificial synthesis is more expensive than that of the natural polymer material, such as chitosan and chitosan oligosaccharide. LMWC-DTX (docetaxel-conjugated chitosan) showed the higher antitumor efficacy but much lower toxicity in comparation with the parent docetaxel (Lee, 2009). Here, chitosan is a natural polysaccharide with low toxic, biocompatible, and biodegradable. Currently, chitosan and its derivatives have been widely explored as vehicles for drug delivery to improve the bioavailability of drugs and reduce adverse drug reactions.

Herein chitosan oligosaccharide (CSO), a low molecular weight chitosan, was utilized as a new delivery platform to form celastrol-conjugated chitosan oligosaccharide (Cel-CSO). There are several advantages of this Cel-CSO conjugate, including low toxicity, excellent water solubility and high bioavailability. Here, we describe the preliminary evaluations of Cel-CSO in pancreatic cancer cells and normal or xenograft tumor-bearing mice, aiming to develop a platform technology for the oral delivery of celastrol. Before that, we reported synthesis, characterization, *in vitro* drug release profile, cytotoxicity, hematological toxicity, and antitumor efficacy of Cel-CSO.

## 2. Materials and methods

### 2.1. Materials

Celastrol was obtained from Xi’an Haoxuan Biotechnology Co. Ltd. (Shanxi, China). Chitosan oligosaccharide (CSO, 1–3 kDa) was purchased from Shanghai Aladdin Biochemical Technology Co., Ltd. (Shanghai, China). Other chemical reagents were purchased from commercial suppliers without purification for the next experiment. Human pancreatic cancer cells (BxPC-3) and hepatic cells (HL7702) were obtained from the American Type Culture Collection (VA, USA) and were cultured according to the instructions. BALB/c (nude) mice (20–22 g) were obtained from the Huaxing laboratory animal farm (Production license NO: 20190002, Zhengzhou, China). All animal studies were performed in accordance with the guidelines of the Institute’s Animal Care and Use Committee in Henan University of Chinese Medicine.

### 2.2. Synthesis and characterizations of Cel-CSO conjugate

The solution DMSO (3 mL) containing Celastrol (45 mg, 0.10 mmol), EDC (28 mg, 0.15 mmol), and HOBt (20 mg, 0.15 mmol) was vigorously stirred for 6 h at room temperature. The reaction mixture was gradually added to the solution of DMSO (5 mL) containing CSO (300 mg). After stirring for 24 h at room temperature, the resulting mixture was diluted by distilled water (5×) and dialyzed against redistilled water using a dialysis membrane (cut off: 500 Da), and then lyophilized into buff solid. The product Cel-CSO was characterized using FT-IR spectrum.

The dried Celastrol (2 mg), CSO (2 mg), Cel-CSO (2 mg) and Cel + CSO mixture (10% Cel, 2 mg mixture) were mixed with the dried KBr, respectively. The mixed samples were pressed into slice. The light transmittance was measured on the attenuated total reflection Fourier transform infrared spectroscopy (Spectrum 100, PerkinElmer Company Ltd., USA). All infrared spectra were obtained at 16 scans with a resolution of 0.1 cm^–1^ at room temperature. The procedure used for baseline correction was in the order of ‘Automatic Correction’ in FT-IR spectrum application software.

### 2.3. Properties studies

10 mg of the compounds (Cel-CSO, Celastrol) were added in 1.0 mL of double distilled water. The mixture was vortexed for 5 min, sonicated for 2 min, and centrifuged at 14,000 rpm for 10 min. The supernatant was quantified by UV spectrophotometer.

The *in vitro* stability and drug release of Cel-CSO were measured in the simulated gastric fluid (SGF; 0.32% pepsin, pH 1.2), simulated intestinal fluid (SIF; 1% pancreatin, pH 7.5), cell culture medium (10% FBS), and mouse plasma at 37 °C for 24 h. Cel-CSO (40 mg) was dissolved in 10 mL of each solution. Then, a total of 200 μL of release solution was collected and replaced with an equal volume of above fresh solution at predetermined time interval. The released Celastrol was subsequently extracted by 1 mL of dichloromethane and was quantified by HPLC on a C_18_ column (4.6 × 250 mm, Agilent) with Methanol/1% acetic acid (87:13, 1 mL/min). The retention time was 15 min.

### 2.4. *In* vitro *cytotoxicity study*

Human pancreatic cancer cell (BxPC-3) and hepatic cell (HL7702) were used to evaluate the cytotoxicity of Cel-CSO and Celastrol. The cells were incubated in cell culture medium containing Celastrol (31.25–4000 nmol/L with 1‰ DMSO) or Cel-CSO (at equivalent concentration of Celastrol) for 24 h and 48 h, respectively. After incubation, the viabilities of cells were evaluated by MTT assay. The optical density of the solution was measured by a microplate reader (Thermo, USA) under 490 nm absorbance values. The IC_50_ values (half-inhibition concentration) of Cel-CSO and Celastrol on cells were calculated by GraphPad Prism7 software.

### 2.5. Analysis of apoptosis

Bxpc3 cells were cultured with Celastrol (1–4 μM) or Cel-CSO (at equivalent concentration of Celastrol) for 24 h. The cells were harvested and rinsed with PBS (3×). Then the cells were resuspended in 500 μL of binding buffer with 10 μL of PI and 5 μL of Annexin V-FITC for 5 minutes. The apoptosis rate in various groups was assayed by flow cytometer (Beckman Coulter, USA).

### 2.6. Wound closure assay

Bxpc3 cells were seeded in a 6-well plate for 100% confluence in 24 hours. In a sterile environment a 200 μL pipette tip was used to make a vertical wound down through the cell monolayer on the top of the tissue culture plate. The culture media and cell debris were carefully removed and slowly added enough basal medium containing Celastrol (1 μmol/L) or Cel-CSO (at equivalent Celastrol of 1, 2, 4 μmol/L) against the well wall to cover the bottom of the well to avoid detaching additional cells. At the end of 0 and 24 h, the plate was taken from the incubator and placed it under an inverted microscope to take a snapshot picture and to check for wound closure.

### 2.7. In vivo toxicity study

The *in vivo* toxicities of Cel-CSO and Celastrol were measured by using normal BALB/c mice. The mice were randomly divided into seven groups (*n* = 6) and administrated orally with Celastrol (2, 4, and 6 mg/kg), Cel-CSO (at equivalent Celastrol of 2, 4, and 6 mg/kg), or saline (Control) every day. The fatality rate and body weight were recorded daily and the blood samples were collected from eyes at the end of experiment.

### 2.8. In vivo antitumor activity

The BxPC-3 tumor-bearing mice were prepared by the previously reported method with some modifications (Zeng et al. 2020). Briefly, BxPC-3 cells (1 × 10^7^, 100 μL) were firstly implanted subcutaneously into right flanks of nude mice. And secondarily, the solid tumor (300–400 mm^3^) were cut into pieces (10 mm^3^) and re-implanted subcutaneously into other nude mice. When tumor node grew up to 100 mm^3^, the mice (*n* = 5) were administrated orally with saline, Celastrol (1 mg/kg) and Cel-CSO (as 1, 2, and 4 mg/kg of Celastrol) every two days, respectively. Tumor volume and body weight were monitored every other day. Finally, the mice were sacrificed according to institutional guidelines at the end of experiment. Subsequently, the tumors, hearts, kidneys, livers, spleens, and lungs were collected and stored at –80 °C.

### 2.9. Histological examination

The tumors, kidneys, livers, hearts, spleens, and lungs of mice were fixed with 4% formalin. After decalcification with 10% EDTA, dehydration with gradient alcohol, paraffin embedding, and pathological section, hematoxylin and eosin (HE) staining were performed for histological examination.

### 2.10. Tunel assay

Apoptosis in tumors was detected by TUNEL kit (Servicebio Corporation, Wuhan, China). Paraffin sections of tumors were deparaffinized with xylene and rehydrated in a series of graded ethanol. Proteinase K solution (10 mg/mL) was used for cell membrane perforation for 30 min at room temperature. Afterwards, the slides were treated in 3% H_2_O_2_/methanol solution for 20 min to eliminate endogenous peroxidase activity. The sections were rinsed with PBS (3×), and then incubated for 1 hour with a mixture of terminal deoxynucleotidyl transferase (TdT) and DIG-dUTD at 37 °C. The sections were thoroughly washed, and covered with Streptavidin-HRP for 30 min in 37 °C, followed by coloration with DAB solution. Finally, hematoxylin was added to stain the nucleus, and the apoptotic cells were stained brown.

### 2.11. Data analysis

All data were expressed as the mean ± SD. Statistical analysis was performed by a two-tailed student’s *t*-test by using SPSS 19.0 statistical package. *p* < .05 were considered statistically significant.

## 3. Results and discussion

### 3.1. Synthesis and characterization of Cel-CSO

**Scheme 1.**
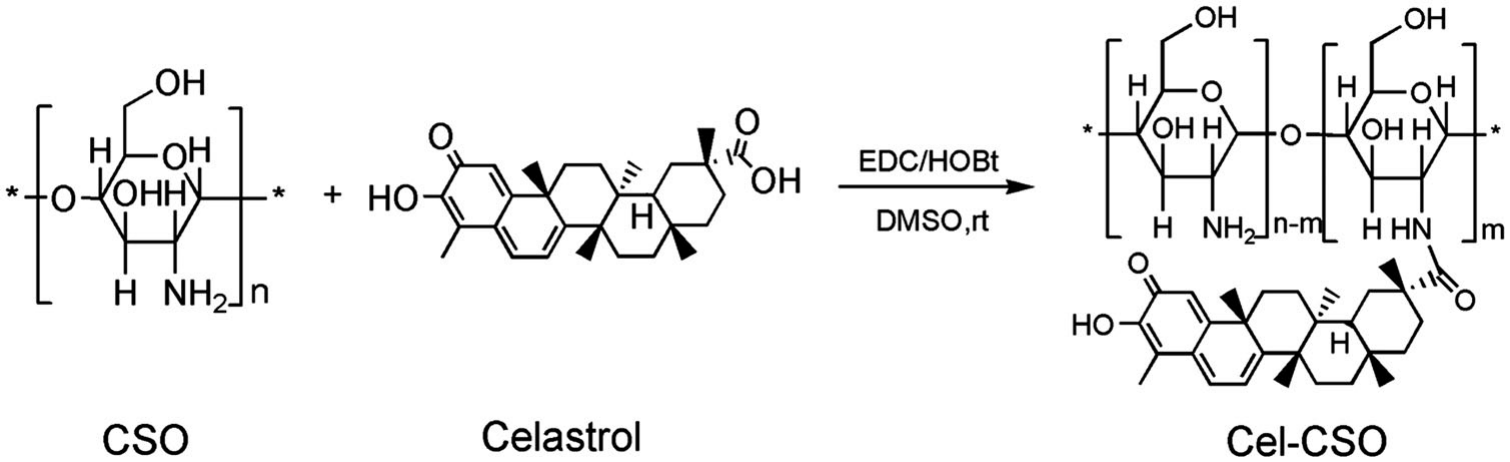
Synthetic procedure of Cel-CSO conjugate.

**Figure 1.**
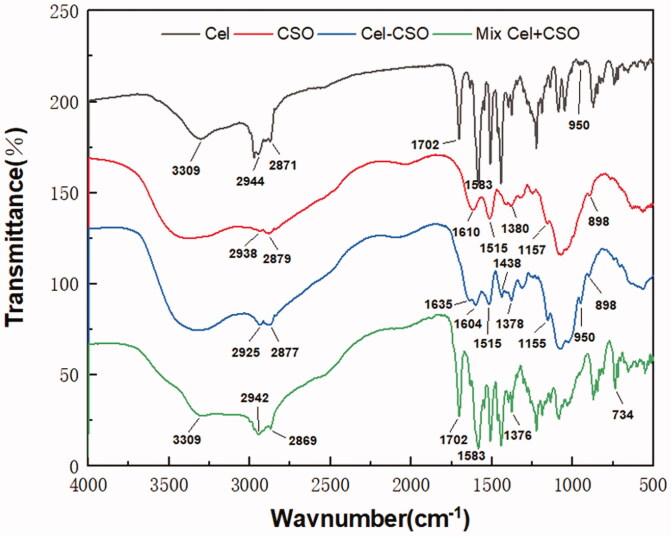
IR spectra of Celastrol, CSO, Cel-CSO conjugate, and the mixture (Cel + CSO).

Cel-CSO was synthesized through covalent bonding between Celastrol and chitosan oligosaccharide (Scheme 1) that could be cleaved to release Celastrol under the physiological environment. The structure of Cel-CSO was confirmed by FT-IR spectroscopy (Figure 1). The strong absorption peak of Cel-CSO at approximately 1635 cm^–1^ was corresponded to the C = O stretching of amide group, whereas the absorption peak at 1702 cm^–1^ (the C = O stretching of fatty acids) disappeared in FTIR spectra of Cel-CSO, demonstrating the successful introduction of amide groups (Figure 1). The ultraviolet spectrum analysis of the conjugate showed that Cel-CSO contained ∼10 wt % Celastrol and had much greater water solubility (18.6 mg/mL) than Celastrol. This indicated that the chitosan oligosaccharide effectively improved water solubility of Celastrol.

**Figure 2.**
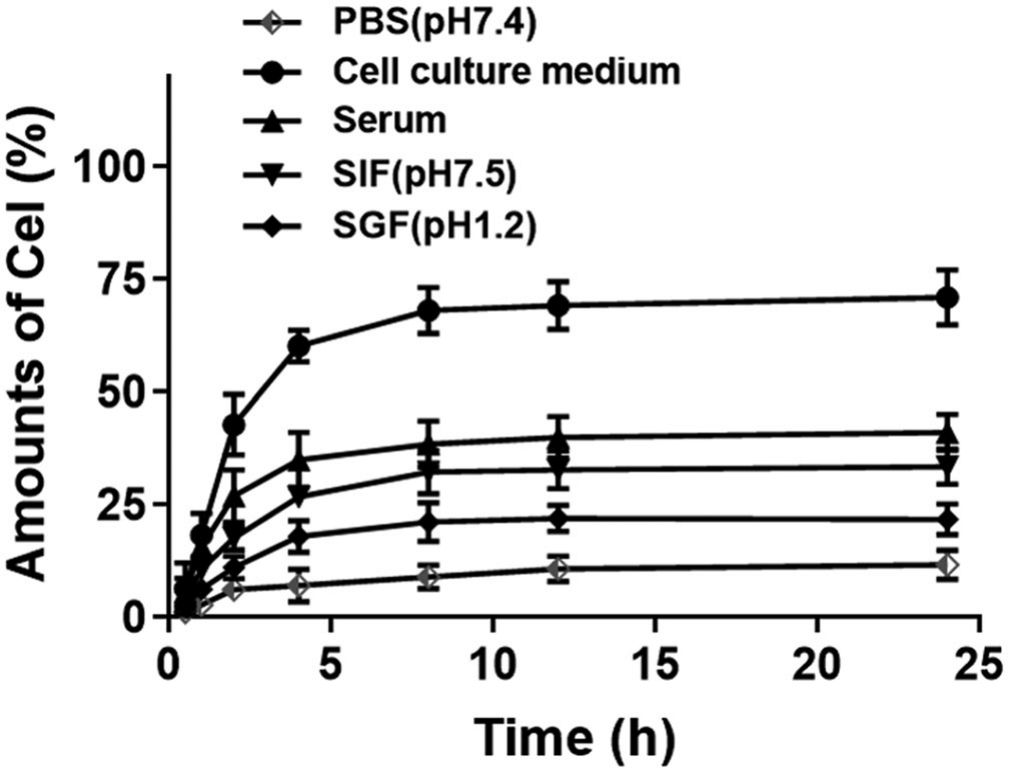
The controlled release manner of Cel-CSO after incubation in PBS (pH 7.4), cell culture medium (10% FBS), serum, SIF (simulated intestinal fluid, pH 7.5), and SGF (simulated gastric fluid, pH 1.2) (*n* = 3).

The drug release analysis of Cel-CSO was used to describe the controlled release ability and stability of the conjugate in SGF and SIF, mouse serum, cell culture medium, respectively (Figure 2). The HPLC analysis showed that the retention time of Celastrol released from Cel-CSO was consistent with that of the native Celastrol, indicating that the amido bonds between Celastrol and CSO were cleaved by some kind of enzymatic attack in those solutions. The low concentrations of Celastrol released from Cel-CSO were observed at 24 h post incubation in PBS (11.69%), SGF (21.75%), SIF (33.45%), and Serum (41.09%), respectively. Approximate 18% of Celastrol was released from Cel-CSO at 4 h post incubation in SGF, while over 70% drug release were completed at 24 h post incubation in cell culture medium. The results indicated that Cel-CSO is relatively stable in SGF with gastric acid and propepsin, while it is very easy to hydrolyze in cell culture medium with complex components, including serum, vitamins, amino acids, enzymes, etc. Considering the transit time in stomach (about 3 h), Celastrol release in SGF would seem insubstantial. Overall, the drug release analysis indicated clearly that most of Celastrol release did not take place under gastric environment, but might mainly happen during or after uptake by the intestine and transport to the blood capillary.

### 3.2. In vitro *cytotoxicity of Cel-CSO*

**Figure 3.**
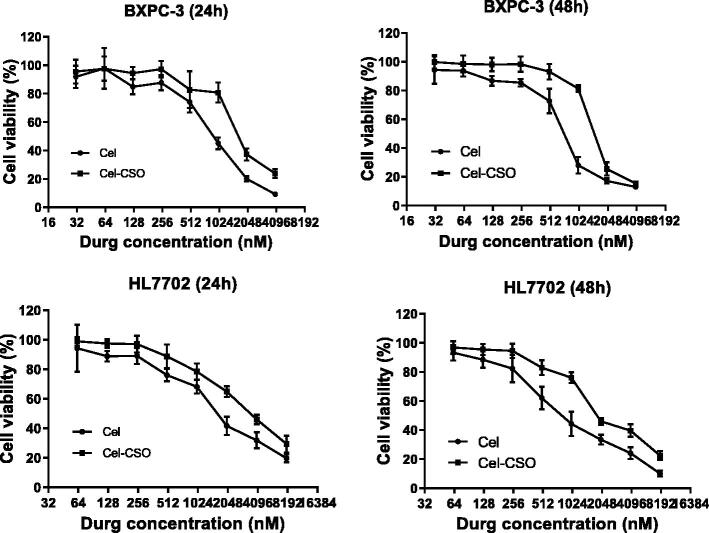
The cytotoxicity of free Celastrol or Cel-CSO against BxPC-3 and HL7702 cells.

The *in vitro* cytotoxicity of Celastrol and Cel-CSO was investigated on BxPC-3 and HL7702 cells at 24 and 48 hours (Figure 3). The IC_50_ values of Celastrol and Cel-CSO in BxPC-3 groups were 929.4 ± 87.58 vs 1436.0 ± 73.40 nmol/L for 24 h (*p* < .01), and 676.8 ± 76.91 vs 1325.0 ± 85.30 nmol/L for 48 h (*p* < .001), respectively (Table 1). Likewise, the IC_50_ values of Celastrol and Cel-CSO in HL7702 groups were 1490.0 ± 80.84 vs 3233.0 ± 98.13 nmol/L for 24 h (*p* < .001), and 793.7 ± 97.84 vs 1647.0 ± 80.55 nmol/L for 48 h (*p* < .001), respectively (Table 1). The Cel-CSO formulation demonstrated nearly half activity loss for inhibiting tumor cells but more than 2-fold hepatotoxicity decrease, which could be regarded as the gradual release of celastrol from Cel-CSO through cleavage of the amido bond between Celastrol and CSO. The results indicated that Cel-CSO can maintain anticancer activity, reduce hepatotoxicity and relieve other side effects in normal organs.

**Table 1.** *In vitro* effect of Celastrol and Cel-CSO (equal to Celastrol concentration) on BXPC-3 and HL7702 cells.

Exposure Time (h)	BXPC-3 (IC_50_, nmol/L)		HL7702 (IC_50_, nmol/L)	
	Cel	Cel-CSO	Cel	Cel-CSO
24	929.4 ± 87.58	1436.0 ± 73.40**	1490.0 ± 80.84	3233.0 ± 98.13^###^
48	676.8 ± 76.91	1325.0 ± 85.30***	793.7 ± 97.84	1647.0 ± 80.55^###^

**p < .01, ***p < .001, vs Celastrol group in BXPC-3; ^###^*p* < .001, vs Celastrol group in HL7702.

### 3.3. Cel-CSO decreased celastrol-induced apoptosis

**Figure 4.**
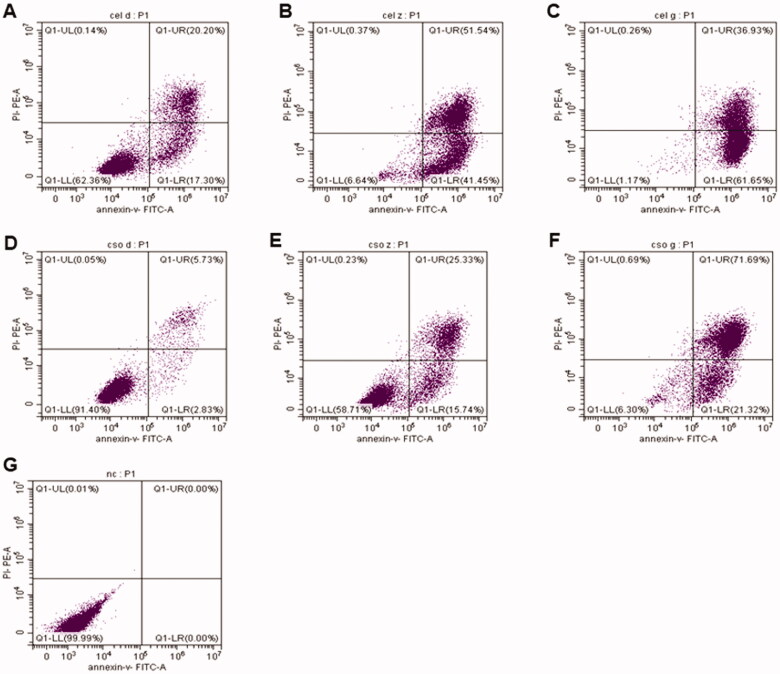
The BxPC-3 cell apoptosis induced by Celastrol (A–C) and Cel-CSO (D–F) detected by flow cytometric method. The concentrations of Celastrol or Cel-CSO (equal to Celastrol concentration) are 0 μmol/L (G), 1 μmol/L (A,D), 2 μmol/L (B,E), 4 μmol/L (C,F), respectively.

The effect of chitosan oligosaccharide as a drug delivery system on Celastrol-induced apoptosis in human pancreatic cancer cell was detected by using a flow cytometry (Figure 4). Compared with the control group, the early apoptosis rates gradually increased from 17.3% to 41.45%, to 61.65%, and the total apoptosis rates increased from 37.5% to 92.99%, to 98.58% after treatment with 1 μmol/L, 2 μmol/L, and 4 μmol/L of Celastrol for 24 h, respectively (Figure 4(A–C)). The results suggested that Celastrol caused a significantly increase in BxPC-3 cell apoptosis in a dose-dependent manner. After incubation with Cel-CSO for 24 hours, the apoptosis rates of BxPC-3 cells were increased from 2.83% to 15.74%, to 21.32% (at early apoptosis stage), and increased from 8.56% to 41.06%, to 93.01% (at whole apoptosis stage) (Figure 4(D–F)). The data revealed that under the same conditions the apoptotic cells were less in Cel-CSO groups than those in Celastrol groups. The results indicated that chitosan oligosaccharide might relieve the Celastrol-induced apoptosis of BxPC-3 cells by controlled release of Celastrol.

### 3.4. In vitro *anti-metastatic effect*

**Figure 5.**
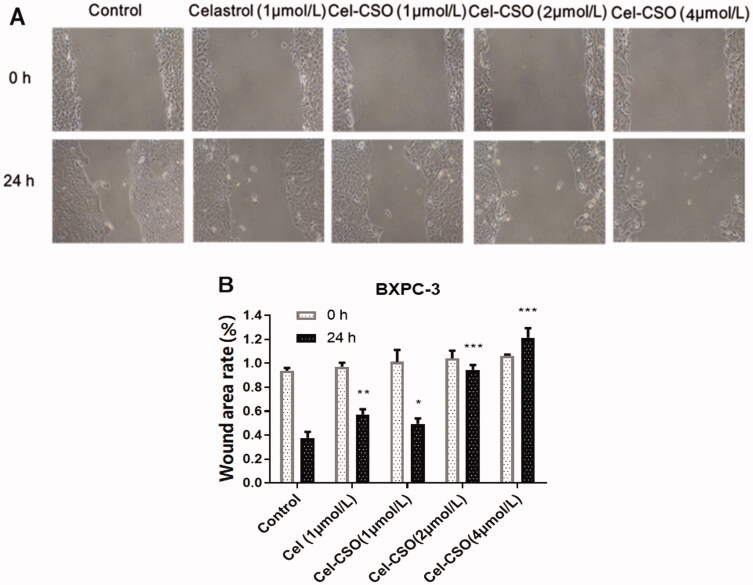
(A) Representative picture of the extent of healing when treated with different formulations for 24 h. (B) Wound area rate of each group for 24 h (*n* = 3). *p < .05, **p < .01, ***p < .001, vs Control group.

The transwell cell migration and invasion assay was performed using BxPC-3 cells, and then the wound closure was recorded using the inverted microscope. Eight pictures at 0 and 24-hour time points are shown in Figure 5(A). The width of the scratched wound produced in the cell monolayer was clearly observed, and the cell uncovered area was calculated (Figure 5(B)). The Figure 5 showed that the closure of the scratched wound could be inhibited by Celastrol and Cel-CSO in a concentration-dependent manner for 24 h. Moreover, 1 μmol/mL of Cel-CSO as well as Celastrol showed a slight inhibition against cell migration and invasion compared with the control group (*p* < .05, *p* < .01). Cel-CSO of 2 and 4 μmol/mL was more efficient in inhibiting cell migration and invasion on the scratched wound (*p* < .001).

### 3.5. In vivo *toxicity of Cel-CSO*

**Figure 6.**
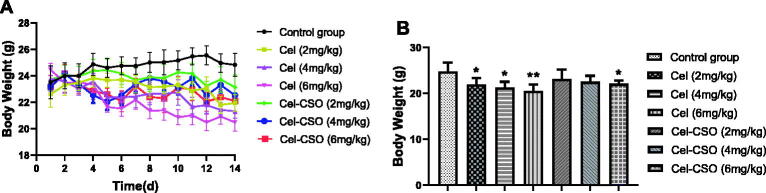
*In vivo* toxicity of Cel-CSO: (A) Monitoring of body weight; (B) Body weight at the end of the experiment. BALB/c mice were administrated with saline, Celastrol (2, 4, 6 mg/kg, p.o.) and Cel-CSO (as Celastrol). *p < .05, **p < .01, vs Control group.

*In vivo* toxicities of Celastrol and Cel-CSO were investigated in normal mice that were assessed by monitoring the body weight and hematological toxicity. All of the administered groups showed obvious body weight loss compared with the control group, while the Celastrol groups demonstrated lower body weight than the Cel-CSO groups at the same concentrations (Figure 6(A)). At the end of the experiment, Cel-CSO groups at the dose of 2 and 4 mg/kg showed no significant differences in body weight compared with control group (*p* > .05), whereas Celastrol group at the dose of 6 mg/kg demonstrated 17.4 ± 2.10% of body weight loss (*p* < .01) (Figure 6(B)). The results indicated that Cel-CSO significantly protected mice from the Celastrol-induced weight loss.

**Figure 7.**
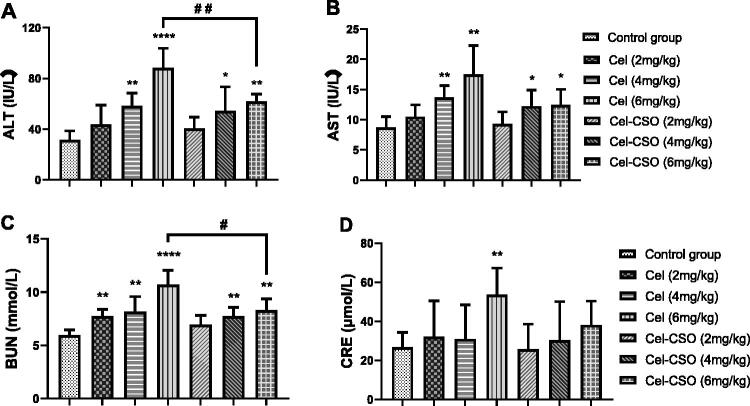
Biochemical parameters of Celastrol and Cel-CSO treated model animals. *p < .05, **p < .01, ****p < .0001, vs Control group; ^#^*p* < .05, ^##^*p* < .01, vs Celastrol (6 mg/kg) group.

Celastrol group after administered at a dose of 6 mg/kg displayed obvious elevation of both ALT and AST in comparison with control group, indicating the occurrence of liver injury (*p* < .01, Figure 7(A,B). However, Cel-CSO groups all showed no significant increase of both ALT and AST. After administered at a dose of 6 mg/kg, Cel-CSO group showed an obvious reduction of both ALT and AST compared with Celastrol group (*p* < .01), which was still normal for hepatic metabolic function. Significant increase of BUN and Crea indicated the kidney dysfunction including dilation of kidney proximal tubules (Figure 7(C,D)). Celastrol at a dose of 6 mg/kg caused higher BUN and Crea level in mouse serum than the Cel-CSO conjugate at the same dose (*p* < .05), while the Cel-CSO conjugates of 2, 4 and 6 mg/kg all had only slight effects on BUN and Crea. The results suggested that the Celastrol treatment was associated with nephrotoxicity and hepatotoxicity. Furthermore, the toxicity of Celastrol increased in dose-dependence manner. The Cel-CSO group demonstrated a slight increase in AST, ALT, BUN and Crea levels, indicating that the incidence of Cel-CSO induced kidney and liver toxicity is very low.

### 3.6. Antitumor activity of Cel-CSO

**Figure 8.**
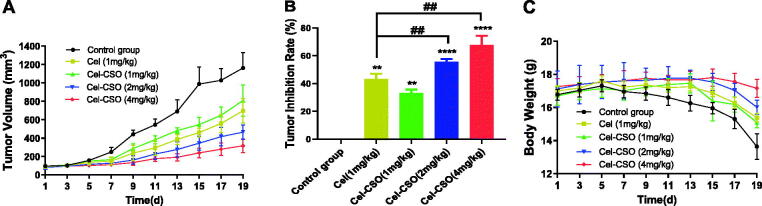
Efficacy and tolerability of Celastrol and Cel-CSO in a mouse model bearing human pancreatic cancer with oral administration every two days. (A) BxPC-3 solid tumor volume over time; (B) the tumor inhibition index at the end of experiment; (C) mouse body weight over time. Tumor inhibition rate (TIR) = [(tumor volume of control group – tumor volume of drug group)/tumor volume of control group] × 100%. **p < .01, ****p < .0001, vs. Control group; ^##^*p* < .01, vs. Celastrol group (1 mg/kg).

The antitumor efficacies of Cel-CSO on human pancreatic carcinoma xenograft models were assessed by monitoring body weight and tumor volume (Figure 8). Compared with control group, the high- and medium-dose Cel-CSO significantly inhibited the tumor growth at the tumor-inhibition rate (TIR) of 67.83 ± 5.31% and 55.69 ± 1.63% (*p* < .0001) for 19 days, respectively, which were better than that of Celastrol (1 mg/kg) at TIR of 43.36 ± 2.87% (*p* < .01) (Figure 8(A,B)). The low-dose Cel-CSO showed the same antitumor efficacy as Celastrol at TIR of 33.09 ± 2.05%. The body weight can reflect the complicated effects from drug toxicity and tumor progression. The control group showed a significant weight loss for up to 19 days, which indicated that the tumor progression caused mainly weight loss (Figure 8(C)). The low-dose Cel-CSO group showed slight difference of weight loss in comparison to Celastrol group (*p* < .05). However, the high- and medium-dose Cel-CSO groups demonstrated a slight weight increase for the first two weeks, following a weight loss, which indicated the antitumor efficacy and toxicity of Cel-CSO were correlated with the dosage. The above results suggested that Cel-CSO was comparably effective in inhibiting tumor growth but had lesser toxic effect than Celastrol. We thought that the controlled release of Cel-CSO promoted the circulation time of Celastrol *in vivo*, which could improve the antitumor efficacy and biological safety of Cel-CSO.

**Figure 9.**
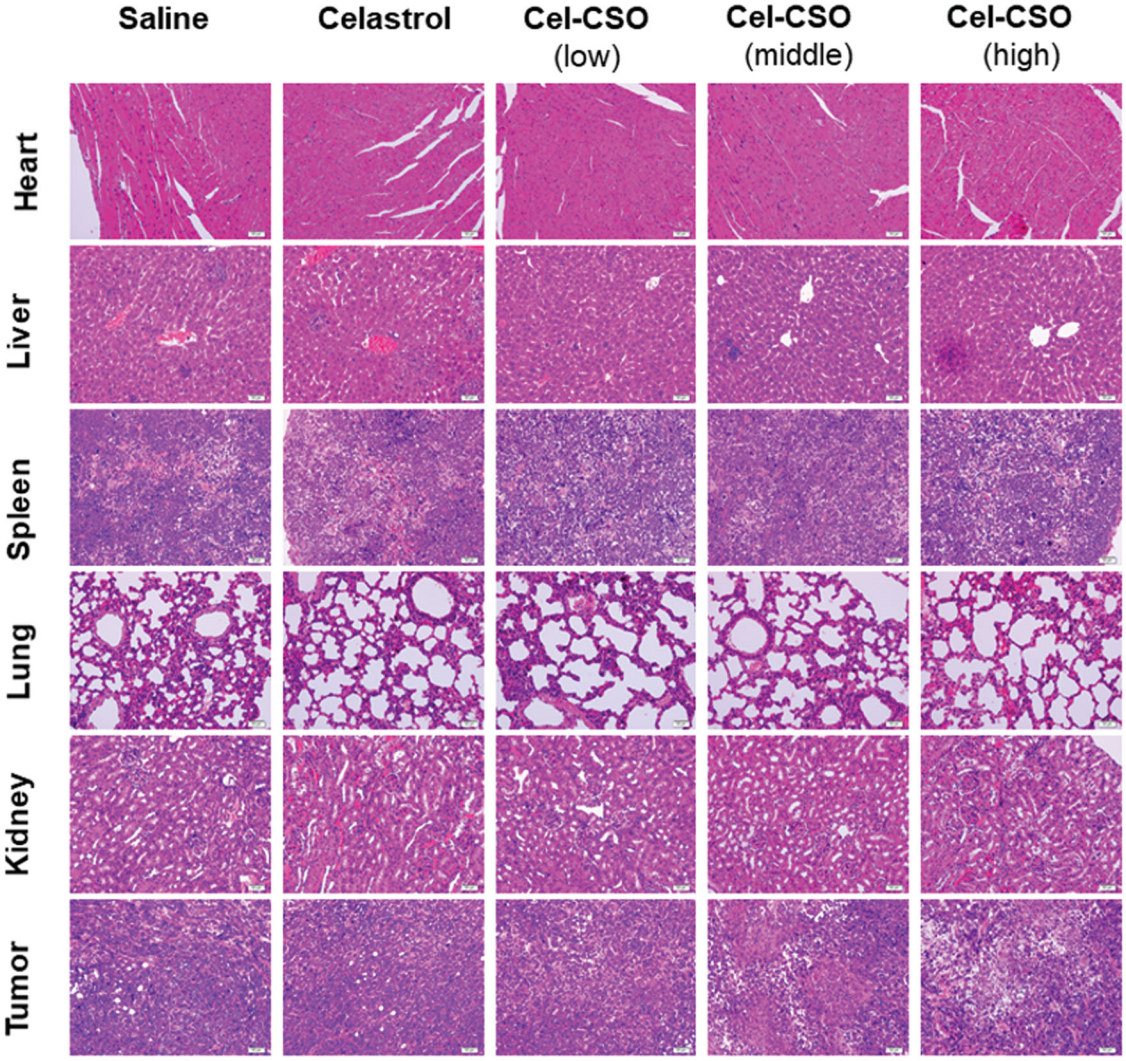
Histological staining of organs and tumor following oral administration of Celastrol formulations (×200).

Figure 9 demonstrated histological micrographs of tumor tissues, livers, hearts, lungs, spleen and kidneys of the tumor-bearing mice treated with saline, Celastrol, and Cel-CSO, respectively. The saline group exhibited dense tumor cells with large and irregular nucleus, obvious nuclear division and negligible apoptosis levels. Compared with the saline group, the administration groups showed some degree of inhibitory effect on tumor. The tumor tissues in the Cel-CSO group showed a lot of cell necrosis, apoptosis, and scattered focal tumor cell nests arranged in gland tube-like. The high- and medium-dose Cel-CSO groups showed not only low toxicity of tripterine to liver, hearts, lungs, spleen and kidney but also superior anticancer efficacy compared to Celastrol group, which should be attributed to CSO polymer provide Cel-CSO an approach to achieve controlled release ability of Celastrol and water solubility. Histopathological studies showed fatty degeneration in the hepatocytes and obvious kidney proximal tubular dilation in mice after oral administration of Celastrol (Figure 9). However, there were no significant histopathological changes in the Cel-CSO groups, which was also consistent with the hematological toxicity results as previously mentioned.

**Figure 10.**
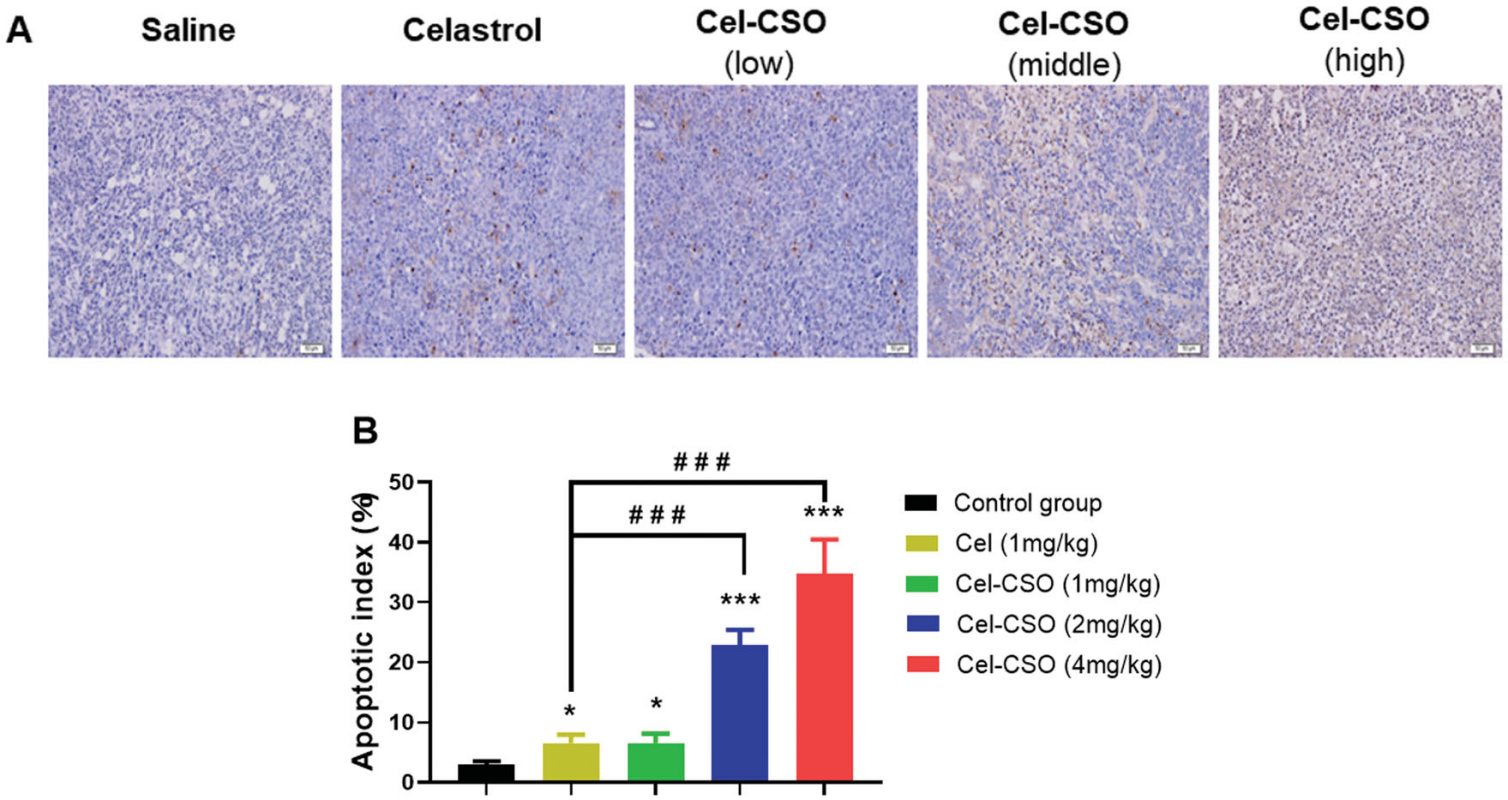
(A) TUNEL assay in tumor tissues (×200); (B) Apoptosis index of tumor cells. *p < .05, ***p < .001, vs. Control group; ^###^*p* < .001, vs. Celastrol group (1 mg/kg). scale bar: 50 μm.

For assessing apoptosis induction *in vivo*, the TUNEL staining on tumor sections in all five groups were performed on the 19th day (Figure 10(A)). Apoptotic cells with brown nuclei were counted under a light microscope in a random field, and the apoptosis index was calculated as a percentage of at least 500 scored cells. The high-dose Cel-CSO caused a significantly higher apoptotic index (34.82 ± 5.64%) in BxPC-3 tumors compared with the medium-dose Cel-CSO (22.81 ± 2.63%), the low-dose Cel-CSO (6.54 ± 1.61%), Celastrol (6.41 ± 1.35%), and control (2.93 ± 0.62%) (*p* < .001, Figure 10(B)). Here, TUNEL assay revealed that Cel-CSO significantly inhibited BXPC-3 tumors cell growth by inducing apoptosis in a dose-dependent manner, which was consistent with the previous study in pancreatic cancer cells. In our present study, these results revealed that the CSO carrier of Cel-CSO did not significantly decrease Celastrol-induced apoptosis in BxPC-3 tumor tissues but obviously relieved apoptosis in BxPC-3 cells, which indicated that Cel-CSO might be apt to target tumor tissues compared to Celastrol.

## 4. Conclusions

A novel chitosan oligosaccharide prodrug of Celastrol was synthesized and characterized by infrared spectrum analyses. Cel-CSO conjugate showed excellent water-solubility (18.6 mg/mL) compared to the parent Celastrol, which could be out of the toxic cosolvent systems (e.g. Tween 80). Cel-CSO also exhibited comparable antitumor efficacy to Celastrol at the same dose level but much lower toxicities were verified by the cytotoxicity, body weight loss, hematological toxicity and H&E staining of several organs compared to that of Celastrol. The better antitumor activity and lower toxicity of Cel-CSO may be attributable to its excellent water-solubility and controlled release of Celastrol. As a consequence, we consider CSO-based conjugate system may be a useful platform for oral delivery of other poorly water-soluble drugs.
